# Supplementary material for: The interactome of intact mitochondria by cross-linking mass spectrometry provides evidence for coexisting respiratory supercomplexes
Source: Mol Cell Proteomics. 2017 Dec 8;17(2):216–32. doi: 10.1074/mcp.RA117.000470 (PMC5795388; doi:10.1074/mcp.RA117.000470)
Supplement: Supplemental Data [file supp_RA117.000470_133922_0_supp_23978_fzffwf.zip › spectra_annotation/mito_DR_spectra_annotation/499-1-4-1-4-1.pdf]

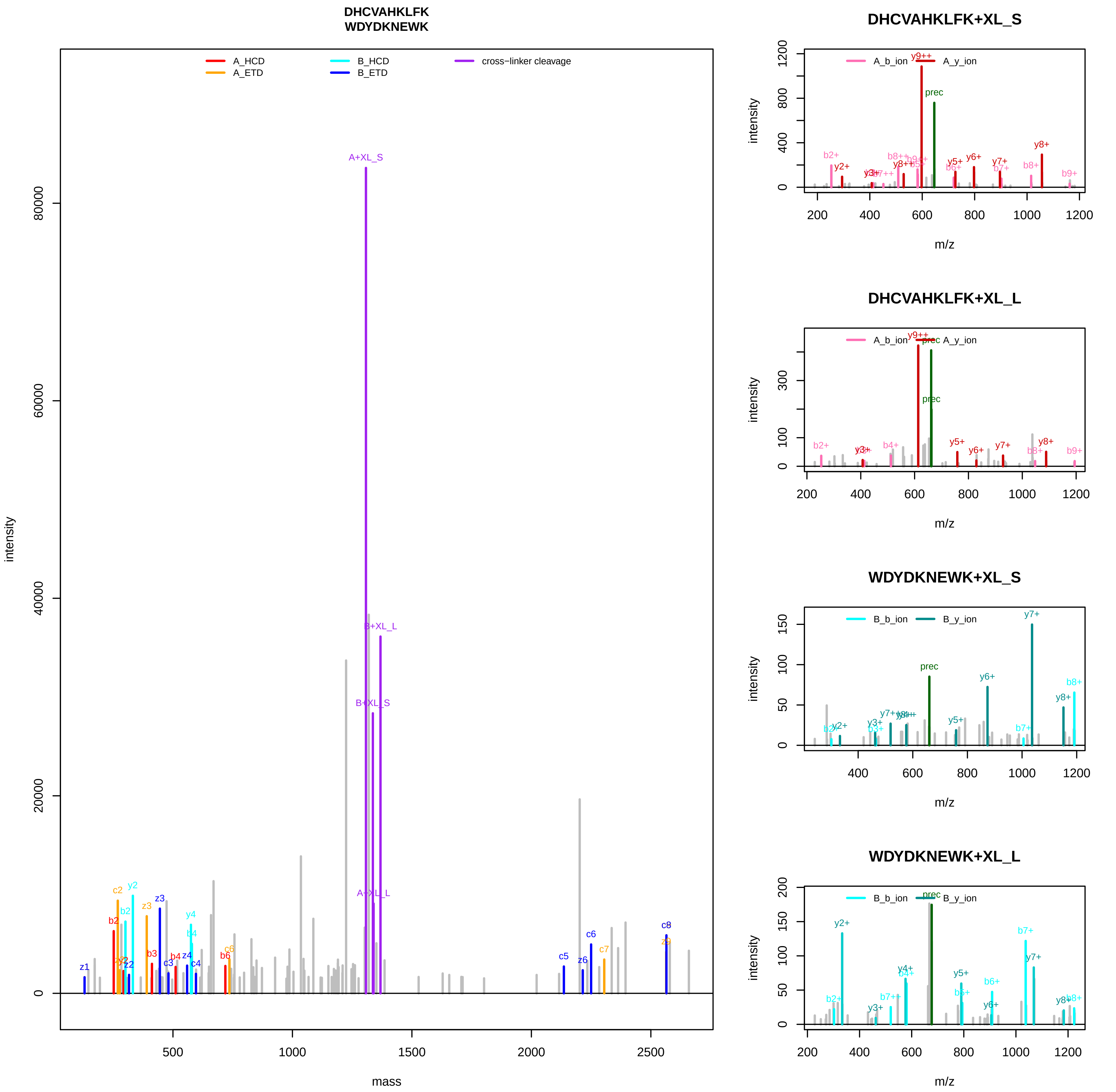
DHCVAHKLFK
WDYDKNEWK
A_HCD
A_ETD
B_HCD
B_ETD
cross−linker cleavage
A+XL_S
B+XL_L
B+XL_S
A+XL_L
intensity
mass
0
20000
40000
60000
80000
500
1000
1500
2000
2500
z1 b2 c2 y2 b2 z3 z3 b3 c3 b4 z4 c4 y4 b4 b6 c6 c5 z6 c6 c7 c8 z9
DHCVAHKLFK+XL_S
DHCVAHKLFK+XL_L
WDYDKNEWK+XL_S
WDYDKNEWK+XL_L
A_b_ion
A_y_ion
B_b_ion
B_y_ion
prec
m/z
intensity
